# Motor Symptom Lateralization Influences Cortico-Striatal Functional Connectivity in Parkinson's Disease

**DOI:** 10.3389/fneur.2021.619631

**Published:** 2021-05-14

**Authors:** Wen Su, Kai Li, Chun-Mei Li, Xin-Xin Ma, Hong Zhao, Min Chen, Shu-Hua Li, Rui Wang, Bao-Hui Lou, Hai-Bo Chen, Chuan-Zhu Yan

**Affiliations:** ^1^Department of Neurology, Research Institute of Neuromuscular and Neurodegenerative Disease, Qilu Hospital of Shandong University, Jinan, China; ^2^Department of Neurology, National Center of Gerontology, Beijing Hospital, Beijing, China; ^3^Institute of Geriatric Medicine, Chinese Academy of Medical Sciences, Beijing, China; ^4^Department of Radiology, National Center of Gerontology, Beijing Hospital, Beijing, China

**Keywords:** Parkinson's disease, functional connectivity, asymmetry, resting-state functional magnetic resonance imaging, striatum

## Abstract

**Objective:** The striatum is unevenly impaired bilaterally in Parkinson's disease (PD). Because the striatum plays a key role in cortico-striatal circuits, we assume that lateralization affects cortico-striatal functional connectivity in PD. The present study sought to evaluate the effect of lateralization on various cortico-striatal circuits through resting-state functional magnetic resonance imaging (fMRI).

**Methods:** Thirty left-onset Parkinson's disease (LPD) patients, 27 right-onset Parkinson's disease (RPD) patients, and 32 normal controls with satisfactory data were recruited. Their demographic, clinical, and neuropsychological information was collected. Resting-state fMRI was performed, and functional connectivity changes of seven subdivisions of the striatum were explored in the two PD groups. In addition, the associations between altered functional connectivity and various clinical and neuropsychological characteristics were analyzed by Pearson's or Spearman's correlation.

**Results:** Directly comparing the LPD and RPD patients demonstrated that the LPD patients had lower FC between the left dorsal rostral putamen and the left orbitofrontal cortex than the RPD patients. In addition, the LPD patients showed aberrant functional connectivity involving several striatal subdivisions in the right hemisphere. The right dorsal caudate, ventral rostral putamen, and superior ventral striatum had decreased functional connectivity with the cerebellum and parietal and occipital lobes relative to the normal control group. The comparison between RPD patients and the controls did not obtain significant difference in functional connectivity. The functional connectivity between the left dorsal rostral putamen and the left orbitofrontal cortex was associated with contralateral motor symptom severity in PD patients.

**Conclusions:** Our findings provide new insights into the distinct characteristics of cortico-striatal circuits in LPD and RPD patients. Lateralization of motor symptoms is associated with lateralized striatal functional connectivity.

## Introduction

Parkinson's disease (PD) is a neurodegenerative disorder commonly seen in the elderly, which manifests as classical motor symptoms such as bradykinesia, rigidity, and resting tremor, together with multiple non-motor symptoms (1). Dopamine deficiency in the striatum is a pathophysiological hallmark in PD and underlies motor and several neuropsychiatric symptoms. The striatum modulates motor activity, cognition, and behavior through multiple cortico-striatal circuits, which involve several striatal subregions (2, 3).

Lateralization is characteristic in PD. Motor symptoms usually present initially in one side of the body, and this asymmetry persists long after both sides show motor dysfunction (4, 5). Lateralization is unique and a clue for differential diagnosis from other neurological disorders presenting as parkinsonism (6). Uneven bilateral deficiency of dopamine in the striatum can explain this motor asymmetry (7–9), but this lateralization affects different cortico-striatal circuits simultaneously and is also related to various non-motor symptoms.

The interaction between cerebral hemisphere dominance and asymmetric brain impairment leads to different neuropsychological profiles in left-onset (LPD) PD and right-onset (RPD) patients. Studies evaluating cognitive function, anxiety, psychosis, and apathy symptoms showed a series of differences between LPD and RPD patients (10–13). Lateralization not only affects clinical profile in PD but also modulates therapeutic responses. In a study by Hanna-Pladdy et al. LPD and RPD patients had different responses to levodopa in attention and even paradoxical responses in verbal memory function (14). Due to different severities of dopamine deficiency in the more affected hemisphere and less affected hemisphere, levodopa may have an ameliorating or overdosing effect to different cortico-striatal circuits (14). Therefore, a better understanding of the effect of lateralization on various cortico-striatal circuits can shed light on a more precise treatment in PD.

Functional magnetic resonance imaging (fMRI) is increasingly used to assess cerebral activity based on the blood oxygen level-dependent (BOLD) effect, which can reflect cerebral blood flow and energy use (15). fMRI can be conducted when the subject is performing a specific task (task-based fMRI) or when the subject lies relaxed [resting-state fMRI (rs-fMRI)] (15). Due to its convenience, rs-fMRI is increasingly used in neurological research. Functional connectivity (FC) is defined as the temporal dependency between different brain regions and is an important approach to analyze rs-fMRI data (15). FC is an ideal technique to explore the impaired cortico-striatal circuits in PD.

There have been several studies showing altered FC between striatum and various brain regions in PD patients, but the seeds used in previous studies varied, and the influence of laterality has rarely been investigated. Some researchers used the nuclei of basal ganglia, such as putamen and caudate as the seeds (16–22); some divided putamen and caudate to the anterior and posterior parts as the seeds (23–29). Others chose representative seeds of the subregions of the striatum (30–35). Most of the studies merged LPD and RPD patients as a single group and compared fMRI data of PD patients with the controls (16, 17, 19, 20, 22, 23, 25, 28, 29, 31, 33–35); some studies only focused on the more severely involved striatum or combined bilateral striatal seeds (27, 30). These approaches cannot discern whether the changed FC was mainly contributed by the LPD or RPD patients or a common impairment shared by LPD and RPD patients.

In the last century, anatomical labeling techniques have demonstrated the existence of parallel cortico-striatal circuits, which are related to motor, cognitive, and limbic functions. In addition, these circuits display rostrocaudal and dorsoventral patterns (36–38). With the advent of functional imaging, studies on the striatum using rs-fMRI have been rapidly increasing. Postuma and Dagher conducted a meta-analysis of positron emission tomography (PET) and fMRI studies. They have revealed that functional imaging can disclose different parallel cortico-striatal circuits and suggested the boundaries between dorsal and ventral caudate and putamen, as well as the boundary between rostral and caudal putamen (39). Furthermore, Di Martino et al. carried out an rs-fMRI study. They integrated the results of the study by Postuma and Dagher and anatomical characteristics of the striatum subregions and defined six seeds in each side of the brain for the rs-fMRI study (3). The seeds chosen by Di Martino et al. can reflect the divergence of these striatal subdivisions and their corresponding FC profiles; these definitions performed well in the following studies (30–35). To date, how lateralization affects different cortico-striatal circuits remains unclear. The present study aimed to utilize rs-fMRI to comprehensively explore the changes of FC of distinct striatal subregions in LPD and RPD patients, in order to reveal the influence of asymmetry on cortico-striatal circuits in PD. The definitions of the seeds are consistent with the studies by Di Martino et al. (3) and Bell et al. (35).

## Materials and Methods

### Participants

Between 2012 and 2014, we enrolled 63 PD patients and 33 age- and sex-matched control subjects without history of neurological or psychiatric disorders. All the participants were right handed and recruited from Beijing Hospital. A movement disorder specialist (W.S. or H.B.C) made the diagnosis based on the UK PD Society Brain Bank diagnostic criteria (6).

We collected demographic and clinical data, including medical history, and physical and neurological examinations from all the subjects. The side of disease onset was identified through retrospective medical records review and patients' reports and supported by neurological examination. The sum of the Unified Parkinson's Disease Rating Scale (UPDRS) part III (including tremor, rigidity, and bradykinesia-related items) score of the right and left limbs was calculated as right and left motor subscores; then we calculated the laterality index by subtracting the left motor subscore from the right motor subscore. Usually, RPD patients had a positive laterality index, and LPD patients had a negative laterality index (40). Patients whose side of onset could not be confirmed concordantly or with bilateral onset were not included. PD patients with dementia, severe head tremor, deep-brain stimulation, substance abuse, head trauma, or other neurological or psychiatric diseases were also excluded.

The MRI scans and clinical and neuropsychological evaluations were performed in a practically defined “off” state, in which the patients had stopped all the antiparkinson agents for ~12 h (overnight). The Hoehn–Yahr staging, UPDRS, Mini-Mental State Examination (MMSE), Hamilton Depression Rating Scale (HAMD), Hamilton Anxiety Rating Scale (HAMA), and Non-Motor Symptoms Questionnaire (NMSQ) were used to measure motor and non-motor symptoms. MMSE was employed to assess cognitive function of the control subjects.

The study was approved by the Ethics Committee of Beijing Hospital, and we conducted the study in keeping with the Declaration of Helsinki. All the subjects signed informed consent prior to participation.

### Image Acquisition

An Achieva 3.0T MRI scanner (Philips Medical Systems, Best, Netherlands) was used for data acquisition. Foam pads were utilized to reduce head motion, and headphones were employed to decrease the scanning noise. The participants were required to lie still with eyes closed, relaxed, and stay awake. A high-resolution T1-weighted anatomical image was acquired using the following parameters: repetition time (TR) = 7.4 ms, echo time (TE) = 3.0 ms, flip angle (FA) = 8°, field of view (FOV) = 240 × 240 mm, matrix size = 256 × 256, voxel dimensions = 0.94 × 0.94 × 1.20 mm, slice thickness = 1.2 mm, and slices = 140. For the rs-fMRI scan, echo-planar imaging (EPI) was performed with the following parameters: TR = 3,000 ms, TE = 35 ms, FA = 90°, FOV = 240 × 240 mm, matrix size = 64 × 64, voxel dimensions = 3.75 × 3.75 × 4.00 mm, slice thickness = 4 mm, slices = 33, and time points = 210 (41).

### rs-fMRI Data Preprocessing

Images were preprocessed using RESTPlus version 1.2 (42), which was based on SPM 12 (http://www.fil.ion.ucl.ac.uk/spm). The preprocessing steps included removing the first 10 volumes to allow for magnetization stabilization, slice-timing to correct for interleaved acquisition, realignment for 3D motion correction, spatial normalization to the Montreal Neurological Institute (MNI) standard space using the co-registered T1 images (43), resampling to 3 × 3 × 3 mm^3^, smoothing with a Gaussian kernel (full-width at half-maximum = 6 mm), time course detrending, nuisance covariate regression [Friston-24 parameters (44) and cerebrospinal fluid and white matter signals], and bandpass filtering (0.01 < *f* < 0.1 Hz). We excluded the subjects whose head movement exceeded 2 mm of displacement or 2° of rotation.

### FC Analysis

**Figure 1:**
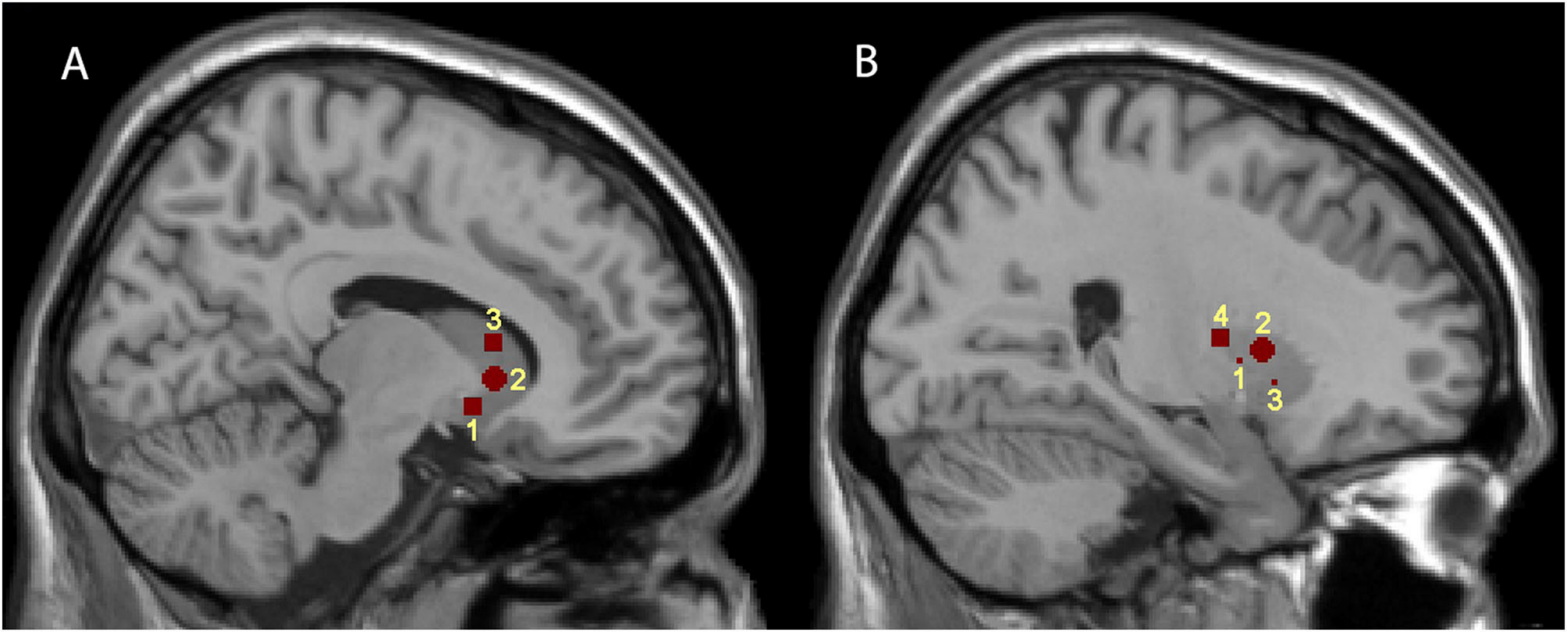
Representations of the seven striatal seed regions. **(A,B)** are sagittal brain views at *x* = 9 and 24, respectively. **(A)** illustrates the positions of the three caudate subdivisions; 1, 2, and 3 represent VSi, VSs, and DC, respectively. **(B)** illustrates the positions of the four putamen subdivisions; 1, 2, 3, and 4 represent DCP, DRP, VRP, and PCP, respectively.

FC maps were obtained using RESTPlus version 1.2, using a seed voxel correlation approach. Because of the dorsoventral and rostrocaudal differences in striatal function and dopamine loss in PD, we chose seeds distributing various locations of the striatum. Di Martino et al. have defined six seeds, including ventral striatum inferior (VSi), ventral striatum superior (VSs), dorsal caudate (DC), dorsal caudal putamen (DCP), dorsal rostral putamen (DRP), and ventral rostral putamen (VRP) (3). We defined six seeds of each hemisphere consistent with Di Martino et al. Since postcommissural putamen (PCP) is especially susceptible in PD and is closely related to motor symptoms (38, 45, 46), we selected a seed of PCP in accordance with Bell et al. (35). The coordinates of the seeds are shown in Table 1, and the positions of the seeds are illustrated in Figure 1. The mean time series of each seed were extracted; then voxel-wise FC analyses were conducted by calculating the temporal correlation between the time series of each seed and those of each voxel within the whole brain. Correlation coefficients were further transformed to z-values via Fisher's z-transformation.

**Table 1:** The co-ordinates of the regions of interest.

**Regions of interest**	**MNI coordinates**		
	**X**	**Y**	**Z**
VSi	± 9	9	−8
VSs	± 10	15	0
DC	± 13	15	9
DCP	± 28	1	3
DRP	± 25	8	6
VRP	± 20	12	−3
PCP	± 26	−4	8

*VSi, ventral striatum inferior; VSs, ventral striatum superior; DC, dorsal caudate; DCP, dorsal caudal putamen; DRP, dorsal rostral putamen; VRP, ventral rostral putamen; PCP, post-commissural putamen*.

### Statistical Analysis

We used SPSS (version 23.0, IBM Corp, Armonk, NY) to analyze demographic and clinical information, as well as extracted FC values. The continuous variables are shown as mean ± standard deviation. Data normality was detected by the Kolmogorov–Smirnov test. One-way ANOVA, Kruskal–Wallis test, *t*-test, or Mann–Whitney *U*-test was employed for between-group comparisons on continuous data when applicable. Fisher's exact test or a chi-square test was used for analyses of categorical variables. *P* < 0.05 was considered statistically significant.

FC analyses were performed using DPABI version 4.2 (47). Analysis of covariance (ANCOVA) was employed to analyze between-group (LPD, RPD, and control groups) differences in FC of the 14 seeds, with age and gray matter density as covariates. The gray matter mask in DPABI version 4.2 was used in the analyses. *Post hoc* pairwise analyses were performed using the least significant difference (LSD) method. Multiple comparisons were corrected according to the Gaussian random field (GRF) theory (voxel level *P* < 0.001; cluster level *P* < 0.05; two-tailed) (48, 49). Cohen's ^2^ was used to evaluate the effect sizes, which was given by DPABI. Pearson's correlation or Spearman's rank correlation was used to investigate the association between the average FC values of significant clusters and clinical and neuropsychological data.

## Results

### Demographic and Clinical Characteristics

Finally, 57 PD patients and 32 controls were enrolled in the analysis, and seven subjects were excluded due to the following reasons: five PD patients and one control participant because of excessive head motion and one PD patient due to unsatisfactory image quality. Thirty PD patients were in the LPD group, and 27 PD patients were in the RPD group.

Table 2 illustrates the demographic and clinical information. The laterality index differed significantly between the two PD groups. Age, sex, and MMSE scores were comparable between the three groups. The LPD and RPD patients had similar mean disease duration, UPDRS score, Hoehn–Yahr staging, HAMD, HAMA, and NMSQ scores.

**Table 2:** Demographic and clinical information of PD patients and controls.

	**LPD**	**RPD**	**Controls**	***P*-value**
Number of subjects	30	27	32	
Age	62.63 ± 8.88	65.85 ± 6.982	62.41 ± 7.07	0.056
Gender (male/female)	14/16	14/13	16/16	0.924
Disease duration	6.80 ± 3.62	6.15 ± 3.59		0.499
Hoehn–Yahr staging	2.13 ± 0.71	2.28 ± 0.67		0.416
UPDRS	49.90 ± 18.82	48.85 ± 12.83		0.809
Laterality index	−5.52 ± 3.57	5.74 ± 3.40		<0.001
MMSE	28.50 ± 1.50	27.56 ± 2.28	27.78 ± 2.25	0.203
HAMD	9.07 ± 5.27	9.56 ± 5.09		0.724
HAMA	9.93 ± 5.04	10.52 ± 6.03		0.691
NMSQ	11.07 ± 5.77	11.56 ± 4.86		0.732

*HAMA, Hamilton Anxiety Rating Scale; HAMD, Hamilton Depression Rating Scale; LPD, left-onset Parkinson's disease; MMSE, Mini-Mental State Examination; RPD, right-onset Parkinson's disease; NMSQ, Non-Motor Symptoms Questionnaire; UPDRS, Unified Parkinson's Disease Rating Scale*.

### Group Differences in FC

ANCOVA and the followed *post hoc* pairwise analyses disclosed significant differences in FC between the two PD groups, as well as between the LPD patients and the controls.

**Figure 2:**
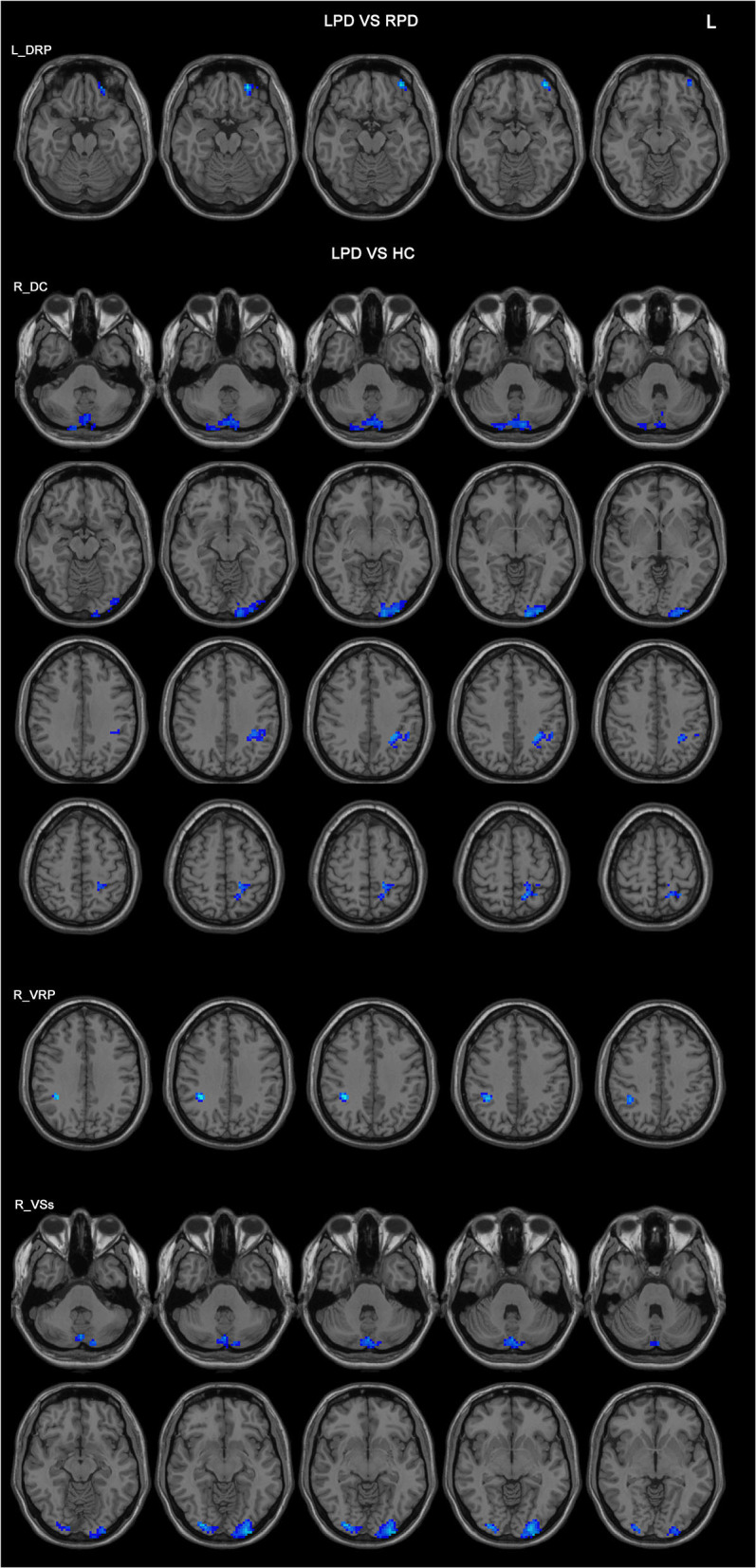
Differences in the FC patterns between LPD and RPD patients and controls. The seed regions are indicated in the left side of the figure. LPD patients had lower FC between the left DRP and the left orbitofrontal cortex compared with RPD patients. LPD patients had lower FCs between the right DC, VRP, VSs, and various brain areas compared with controls. HC, healthy controls; DC, dorsal caudate; DRP, dorsal rostral putamen; L, left side of the brain; LPD, left-onset Parkinson's disease; RPD, right-onset Parkinson's disease; VRP, ventral rostral putamen; VSs, ventral striatum superior.

In the comparison between the LPD patients and the RPD patients, only one seed showed significant difference in FC between the two groups. The LPD patients had lower FC between the left DRP and the left orbitofrontal cortex than the RPD patients (Figure 2 and Table 3).

**Table 3:** Differences in FC among PD patients and controls.

**Seed regions**	**Connected area**	**Peak MNI coordinates**			**Number of voxels**	***T*-value**	**Effect size (Cohen's *f*^***2***^)**
		**X**	**Y**	**Z**			
**LPD < RPD**							
Left DRP	Left orbitofrontal cortex	−36	51	−15	42	−4.22	0.23
**LPD < HC**							
Right DC	Cerebellum posterior lobe	18	−90	−45	231	−4.34	0.21
	Left occipital lobe	−15	−102	−6	186	−4.26	0.23
	Left inferior parietal lobe	−30	−45	39	76	−4.28	0.22
	Left superior parietal lobe	−15	−54	60	65	−4.18	0.24
Right VRP	Right parietal lobe	36	−42	36	50	−4.53	0.26
Right VSs	Cerebellum posterior lobe	0	−75	−39	110	−3.96	0.20
	Left occipital lobe	−30	−99	−9	129	−4.14	0.22
	Right occipital lobe	30	−90	−9	60	−4.08	0.22

*HC, healthy controls; DC, dorsal caudate; DRP, dorsal rostral putamen; LPD, left-onset Parkinson's disease; MNI, Montreal Neurological Institute; RPD, right-onset Parkinson's disease; VRP, ventral rostral putamen; VSs, ventral striatum superior*.

Compared with the controls, LPD patients showed altered FC in three seeds: the right DC, the right VRP, and the right VSs, all in the right side. The aberrant FCs in LPD patients were as follows: (1) decreased FC between the right DC and the cerebellum posterior lobe, the left occipital lobe, the left inferior parietal lobe, and the left superior parietal lobe compared with the controls (Figure 2 and Table 3); (2) decreased FC between the right VRP and the right parietal lobe (Figure 2 and Table 3); (3) decreased FC between the right VSs and the cerebellum posterior lobe, the left occipital lobe, and the right occipital lobe (Figure 2 and Table 3).

### Correlation Analysis

Pearson's correlation or Spearman's rank correlation was used to investigate the relationship between the left DRP–orbitofrontal cortex FC and Hoehn–Yahr staging, contralateral motor subscore of UPDRS part III, laterality index, MMSE, HAMD, HAMA, and NMSQ scores.

**Figure 3:**
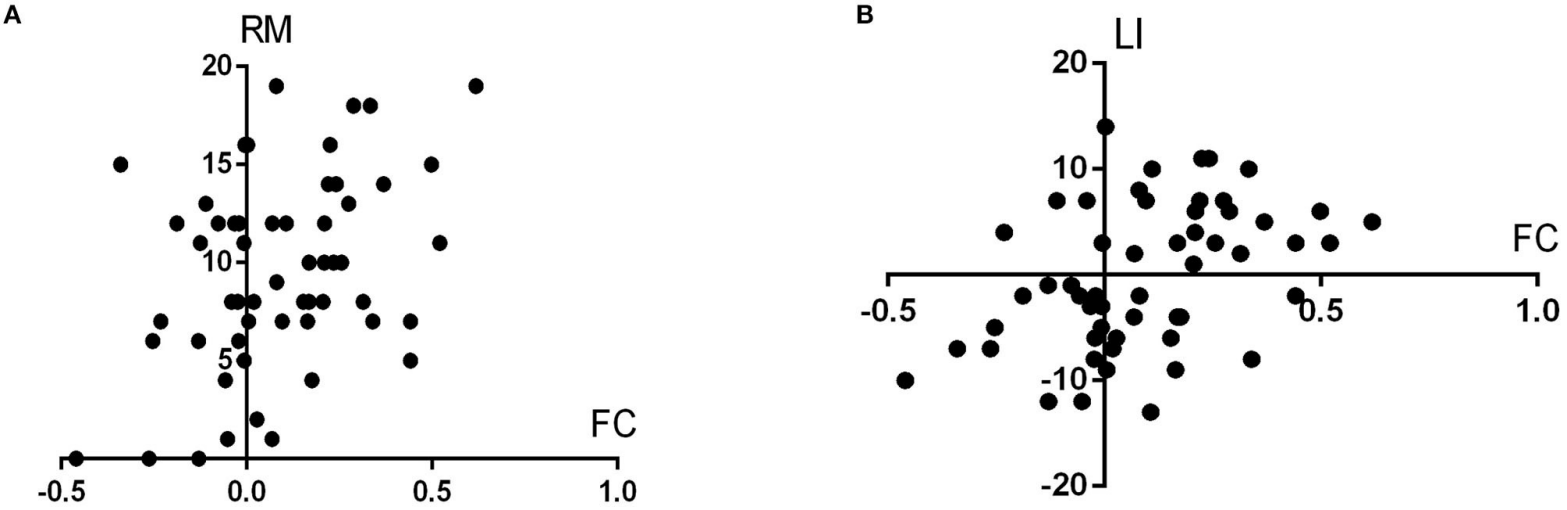
**(A,B)** illustrate the scatterplots showing the associations between the left DRP–orbitofrontal cortex FC and the UPDRS part III score of the right side (RM in the figure, represents right motor score) and laterality index (LI), respectively.

FC between the left DRP and the left orbitofrontal cortex was significantly associated with the right motor subscore of UPDRS part III and laterality index in PD patients (*r* = 0.387 and 0.418; *p* = 0.003 and 0.001, respectively) (Figure 3).

## Discussion

To the best of our knowledge, this is the first study systematically exploring FC related to striatal subregions in LPD and RPD patients separately. We demonstrated that FC between the left DRP and the left orbitofrontal cortex was different between LPD and RPD patients, and LPD patients had a series of differences in FC between various brain regions and the right DC, the right VRP, and the right VSs compared with the controls. The changed FC between the left dorsal rostral putamen and the left orbitofrontal cortex was associated with contralateral motor symptom severity and laterality index.

In healthy subjects, the activity of DRP is predominantly associated with sensorimotor areas (3), but in PD, the specificity of its connectivity is reduced and the FC of DRP extends to the ventromedial prefrontal cortex (3, 31). Our results showed that LPD and RPD patients differed in the FC between left DRP and left orbitofrontal cortex. In addition, the FC between left DRP and left orbitofrontal cortex was significantly associated with the severity of contralateral motor symptoms; the higher the FC, the more severe the contralateral motor symptoms. These results confirm the role of DRP in regulating movement and indicate that the altered left DRP–orbitofrontal cortex FC might be a pathological change in PD. The significant association between left DRP–orbitofrontal cortex FC and laterality index affirms our hypothesis that motor asymmetry can influence cortico-striatal circuits.

It is noteworthy that several aberrant FCs were identified only in LPD patients compared with the controls, and these abnormal FCs all involved the striatal seeds of the more severely impaired hemisphere. This finding corroborates our hypothesis that uneven impairment of the bilateral nigrostriatal function leads to lateralized FC changes in PD. On the other hand, the comparison between RPD patients and the controls obtained no significant difference. These two comparisons indicate that LPD patients might have more severe FC impairments than RPD patients, especially in the right hemisphere. Some clinical observations demonstrated that LPD and RPD patients might have different disease severities and risks of future motor complications and that RPD might be a slightly more benign subtype than LPD (50, 51). A study by Lee et al. compared gray matter volume across controls and LPD and RPD patients. They found several abnormalities of gray matter volume also in the right hemisphere in LPD patients, but they did not identify any significant difference between the two PD groups or between the controls and the RPD patients (52). Two additional MRI studies using structural and functional imaging techniques also showed more impairments in LPD patients than in RPD patients (41, 53). Our findings are consistent with the above studies; LPD patients may have more severe neurodegeneration or less compensation than RPD patients. Maybe a larger sample can better discriminate impaired FC in RPD patients. Additionally, we need to be aware that some controversy exists regarding which type is more susceptible; a study by Baumann et al. showed that RPD patients had a more rapid decline (54). Nevertheless, more clinical and imaging research is needed to clarify the role of laterality in PD.

On the whole, only one different FC was identified between the two PD groups; however, there were much more significant differences in the comparison between LPD patients and the controls. This phenomenon is not uncommon. Some previous studies using structural imaging and fMRI techniques failed to identify significant differences in the direct comparison between LPD and RPD patients, although these two groups showed different patterns of abnormalities compared with the controls (41, 52, 53, 55). In the present study, both PD groups had an average disease duration of more than 6 years and an average Hoehn–Yahr stage higher than 2. At the time of examination, most of the PD patients had bilateral striatal impairments. Although the laterality index showed that there was still obvious asymmetry in the PD patients, the impairments and compensation mechanism are complicated in this stage. The effects of asymmetry might be minor and difficult to detect sufficiently with a relatively small sample size and stringent multiple comparison corrections. Additionally, conflicting results exist on the persistence of laterality in PD; some researches showed a decreased degree of asymmetry with disease progression (5, 56). The laterality of FC might also decrease with disease progression. Maybe future studies recruiting PD patients in an earlier stage can better demonstrate the influence of lateralization on striatal FC.

There has been a variety of abnormal FCs reported in PD, from the early to late stages, but our comparison between RPD patients and the controls attained no significant findings. We need to take the methodological details into consideration. First of all, most of the previous studies combined LPD and RPD patients into a single group. This approach could increase the sensitivity of discovering impaired cortico-striatal FC in PD, particularly those impairments shared by LPD and RPD patients. Dividing the two subgroups according to the side of onset decreases the sample size of each group; this might partially contribute to our negative results in the comparison between RPD patients and the controls. Second, previously, a large number of the FC studies on PD used less strict multiple comparison corrections. To some extent, this might account for the large number of positive findings. This issue was raised by a widely concerned article published by Eklund et al. (57), in which several popular multiple comparison correction approaches had an unsatisfactory performance. For instance, the AlphaSim correction was popular (16, 17, 22, 25, 31, 58) and was not recommended by recent methodological studies (49, 57). Based on these methodological studies, we corrected for multiple comparisons based on GRF theory, with stringent thresholds (voxel level *P* < 0.001; cluster level *P* < 0.05; two-tailed). The stringent thresholds and small sample size may limit the sensitivity to disclose aberrant cortico-striatal FC in RPD patients. Future studies with a larger sample size and strict control for multiple comparisons may better reveal FC impairments in RPD patients. Finally, as we have mentioned, RPD patients may have a better neural reserve and/or greater neural plasticity than LPD patients. The impairment of FC of RPD patients may be milder than that of LPD patients and need a larger sample size to be detected.

Some limitations should be noted. First, the number of participants in this study is relatively small, and only right-handed PD patients were enrolled. Future studies recruiting more subjects and including left-handed PD patients can provide new insights on the topic of lateralization in PD. Second, the enrolled patients underwent chronic dopaminergic drugs, and the medications might interfere with the rs-fMRI results. To control the pharmacological effects, we evaluated the PD patients during the off period. Although the influence of these medications cannot be completely eliminated, this is a commonly used approach and helps compare with similar studies from other researchers. Furthermore, similar alterations of rs-fMRI results in *de novo* PD patients, and off-medication patients have been reported (59). Therefore, the influence of dopaminergic drugs should not be a major concern, and future studies using drug-naïve PD patients can better address this issue. Third, the cognitive function was evaluated with MMSE, which was not fully recommended by the Movement Disorder Society (MDS) task force (60). MMSE has limited coverage of executive function. This is a limitation of the present study. The study was designed in 2011 and conducted between 2012 and 2014. In a review article published in 2007 (61), MMSE was proposed as a level 1 testing for the diagnosis of PD dementia. Therefore, MMSE was used as a screening instrument for cognitive dysfunction in the study. In future studies, we will use the Montreal Cognitive Assessment (MoCA) instead of MMSE. In addition, apathy is an important non-motor symptom in PD, but we did not assess apathy in this study. This insufficiency prevents us from analyzing the relationship between changed FC and apathy.

In conclusion, we found different cortico-striatal FC profiles between LPD and RPD patients and between LPD patients and controls. Lateralization of motor symptoms is associated with lateralized striatal FC. These results emphasize the necessity of separate investigations of the characteristics of brain activities of LPD and RPD patients in future studies using functional imaging modalities.

## Data Availability Statement

The raw data supporting the conclusions of this article will be made available by the authors, without undue reservation.

## Ethics Statement

The studies involving human participants were reviewed and approved by The Ethics Committee of Beijing Hospital. The patients/participants provided their written informed consent to participate in this study.

## Author Contributions

WS, H-BC, and C-ZY conceived and designed the experiments. KL analyzed the fMRI data. MC, C-ML, RW, and B-HL were responsible for the fMRI scans and helped fMRI data analyses. WS, H-BC, and S-HL recruited the subjects. X-XM, HZ, KL, and S-HL collected the demographic, clinical, and neuropsychological information of the subjects. KL and WS wrote the manuscript. All the authors have read, revised, and approved the final manuscript.

## Conflict of Interest

The authors declare that the research was conducted in the absence of any commercial or financial relationships that could be construed as a potential conflict of interest.
